# Supplementary material for: The effectiveness of language nursing intervention on mental health in children with poor language skills
Source: PLoS One. 2024 Nov 1;19(11):e0313095. doi: 10.1371/journal.pone.0313095 (PMC11530037; doi:10.1371/journal.pone.0313095)
Supplement: Attachment — Submitted filename: PONE-D-24-32947_REV.pdf [file pone.0313095.s002.pdf]

Review of the Research Article: "The effectiveness of language nursing intervention on mental health in children with poor language skills"

Overall Assessment:

The article presents a well-structured and comprehensive study investigating the long-term impact of a language nursing intervention on the mental health of children with poor language skills. The authors employed a robust methodology, including a multi-stage, stratified, cluster random sampling design and a follow-up evaluation. The findings suggest that the intervention is effective in improving both language skills and mental health outcomes.

Strengths:

1. **Clear Research Question and Significant Research Problem:** The study clearly articulates its aim to evaluate the long-term impact of a language nursing intervention on mental health.
2. **Rigorous Study Design and Methodology:** The use of an experimental-control group design and long-term follow-up provides a solid methodological framework. The combination of quantitative measures, including language assessments and mental health evaluations, offers comprehensive data that enhances the study's validity and potential generalizability.
3. **Long-term follow-up (approximately 6 years):** The longitudinal design with a follow-up evaluation provides valuable insights into the long-term effects of the intervention and provides insight into lasting effects of early intervention.
4. **Validated assessment tools (DREAM, CES-D, DASS-21):** Using these tools enhance reliability of measurements.
5. **Comprehensive Analysis:** The authors conducted both univariate and multivariate analyses to examine the relationships between variables and mental health outcomes.
6. **Strong Results:** The significant improvement in language skills for the experimental group and the identification of key factors associated with mental health outcomes (such as single-parent family status and participation in the nursing protocol) are clearly mentioned and explained.

Limitations:

1. **Literature Review:** The literature review could be more comprehensive. While it cites relevant studies, it does not adequately explain the gap this research fills compared to previous studies. The authors mention that little research has been done on long-term effects but do not detail what has been done, leaving the gap somewhat vague.
2. **Sample Size and Generalizability:** While the sample size is relatively large, it's important to consider the generalizability of the findings to different populations and cultural contexts as the study focused on one region in China. The study focuses on children from specific public and private kindergartens in Hunan, China. This narrow focus might limit the generalizability of the findings to other regions or countries with different educational or healthcare systems. A section discussing these limitations would enhance the paper.
3. **Questions/Areas for Further Research and future direction:** Consider adding a sentence that connects the limitations with possible areas for future research. Perhaps future direction may ask one or some of the following questions:

   1. What specific components of the nursing intervention were most effective?
   2. How do these findings compare to similar interventions in other cultural contexts?
   3. Are there any gender differences in the long-term outcomes?
   4. What are the potential mechanisms linking early language skills to later mental health outcomes?

In conclusion, this study offers valuable evidence highlighting the long-term benefits of early language interventions on children's mental health and underscores the effectiveness of language-based interventions in enhancing both language skills and mental health outcomes for children with language difficulties. However, it is important to interpret these findings within the context of the study's limitations, acknowledging factors that may influence the results. These findings contribute to a broader understanding of the intricate relationship between language, cognition, and mental well-being, reinforcing the need for integrated approaches in child development and healthcare.

I recommend publishing this paper after careful consideration of the limitations.
