# Supplementary material for: The effectiveness of language nursing intervention on mental health in children with poor language skills
Source: PLoS One. 2024 Nov 1;19(11):e0313095. doi: 10.1371/journal.pone.0313095 (PMC11530037; doi:10.1371/journal.pone.0313095)
Supplement: Attachment — Submitted filename: PLOS ONE 2024 review.docx [file pone.0313095.s001.docx]

The article presents a valuable contribution to the literature on the relationship between language skills and mental health in children. The study design, data analysis, and discussion of findings are generally well-executed. However, there are a few areas where the article could be strengthened.

There are a few minor grammatical or stylistic issues that could be addressed, but they do not significantly impact the overall readability or clarity of the text.

**Introduction**

**Line 35:** The statistic of 17.5% of Chinese children and adolescents with mental disorders could be further contextualized by providing information about the prevalence of mental health disorders in other countries or regions.

**Lines 62-65:** The mention of Qiang (2023) could be strengthened by providing more details about the specific findings of that study regarding the short-term effects of language nursing intervention.

**Methods**

**Line 70:** The phrase "semi-experimental method" could be further clarified to specify the type of semi-experimental design used (e.g., pretest-posttest with control group).

**Lines 73-78:** The description of the multi-stage, stratified, cluster random sampling design is clear and well-explained. However, it might be helpful to add a visual diagram or flowchart to illustrate the process.

While the sample size of 6,820 children is substantial, it might be helpful to provide additional information about the distribution of participants across different demographic variables (gender, socioeconomic status).

If there were any dropouts during the study, it would be important to report the dropout rate and reasons for attrition.

**Procedures**

There are a few minor inconsistencies or typos: For example, in line 93, "questionnaire" should be capitalized as "Questionnaire."

**Line 98:** Consider rephrasing "they took the Raven Standard Progressive Matrices test" to "they were administered the Raven Standard Progressive Matrices test."

**Lines 113-114:** The phrase "from 3-4 to 9-10 years old" could be clarified to "from age 3-4 to age 9-10."

**Line 124:** "four-point Likert scale" could be clarified as "a 4-point Likert scale."

**Statistical analysis**

In addition to statistical significance, it would be beneficial to report effect sizes (e.g., odds ratios) to quantify the magnitude of the relationships between variables.

It would be helpful to provide information about the model fit (e.g., R-squared, Nagelkerke R-squared) to assess the overall performance of the regression models.

It is important to check the assumptions of the logistic regression models (e.g., linearity, multicollinearity) to ensure the validity of the results.

**Results**

While the statistical significance of the findings is clear, reporting effect sizes (e.g., odds ratios) would provide a more complete picture of the magnitude of the relationships between variables.

It would be helpful to discuss the clinical significance of the findings, particularly in terms of the practical implications for intervention and prevention.

**Discussion**

**Paragraphs 1-3:** The discussion of the prevalence of developmental language disorder and its association with mental health challenges is clear and well-supported by literature.

**Paragraphs 4-5:** The discussion of the study's findings regarding the effectiveness of the nursing intervention and the relationship between single-parent families and mental health is well-organized and easy to follow.

**Paragraphs 2-3:** The comparison of developmental language disorder with autism spectrum disorder and ADHD is accurate and provides valuable context.

**Paragraph 4:** The discussion of the limitations of the study is balanced and acknowledges potential areas for future research.

The discussion could be further strengthened by explicitly outlining the practical implications of the findings for clinicians working with children with language disorders.

The limitations could be followed by specific recommendations for future research to address these limitations and further explore the relationship between language skills and mental health.
