# Supplementary material for: The effectiveness of language nursing intervention on mental health in children with poor language skills
Source: PLoS One. 2024 Nov 1;19(11):e0313095. doi: 10.1371/journal.pone.0313095 (PMC11530037; doi:10.1371/journal.pone.0313095)
Supplement: Attachment — Submitted filename: Response to Reviewers.docx [file pone.0313095.s003.docx]

Dear editor

- The “minimal data set” of study is present entirely within manuscript
- The list of authors and affiliations are correct.

Reviewer 1:

the study employs various measures, and it is imperative to address the reliability and validity of these instruments.

Answer: The reliability and validity of the questionnaires were entered in the Procedures section.

demographic backgrounds of the participants are not adequately detailed”

answer: I added table 1.

Given that this is a longitudinal study and that the results suggest that family structure, particularly single-parent status, plays a significant role in the mental health outcomes for children, it is necessary to inquire whether there have been any changes in the marital status of the participants' families over the course of the study. Furthermore, clarification is needed regarding how such changes were addressed in the data analysis.

answer: I added table 1.

Reviewer 2:

The statistic of 17.5% of Chinese children and adolescents with mental disorders could be further contextualized by providing information about the prevalence of mental health disorders in other countries or regions.

Answer: I added to introduction (lines 34-37).

The mention of Qiang (2023) could be strengthened by providing more details about the specific findings of that study regarding the short-term effects of language nursing intervention.

Answer: The type of intervention, its duration and the participants are specified.: lines 64-65.

Line 70: The phrase "semi-experimental method" could be further clarified to specify the type of semi-experimental design used (e.g., pretest-posttest with control group).

Answer: I added. Lines: 84-88.

Lines 73-78: The description of the multi-stage, stratified, cluster random sampling design is clear and well-explained. However, it might be helpful to add a visual diagram or flowchart to illustrate the process.

Answer: I added in study design section.

While the sample size of 6,820 children is substantial, it might be helpful to provide additional information about the distribution of participants across different demographic variables (gender, socioeconomic status).

Answer: I added table 1.

If there were any dropouts during the study, it would be important to report the dropout rate and reasons for attrition.

Answer: In the first stage, some participants did not have written consent. In the follow-up phase, we could not find 15 participants. It is clear in Figure 1.

Procedures
There are a few minor inconsistencies or typos: For example, in line 93, "questionnaire" should be capitalized as "Questionnaire."
Line 98: Consider rephrasing "they took the Raven Standard Progressive Matrices test" to "they were administered the Raven Standard Progressive Matrices test."
Lines 113-114: The phrase "from 3-4 to 9-10 years old" could be clarified to "from age 3-4 to age 9-10."
Line 124: "four-point Likert scale" could be clarified as "a 4-point Likert scale."

Answer: I edit it. Thanks.

In addition to statistical significance, it would be beneficial to report effect sizes (e.g., odds ratios) to quantify the magnitude of the relationships between variables.
It would be helpful to provide information about the model fit (e.g., R-squared, Nagelkerke R-squared) to assess the overall performance of the regression models.
It is important to check the assumptions of the logistic regression models (e.g., linearity, multicollinearity) to ensure the validity of the results.

Answer: I added to results section.

Results
While the statistical significance of the findings is clear, reporting effect sizes (e.g., odds ratios) would provide a more complete picture of the magnitude of the relationships between variables.
It would be helpful to discuss the clinical significance of the findings, particularly in terms of the practical implications for intervention and prevention.

Answer: I added to results section.

Reviewer3: The literature review could be more comprehensive. While it cites

relevant studies, it does not adequately explain the gap this research fills compared to

previous studies. The authors mention that little research has been done on long-term effects

but do not detail what has been done, leaving the gap somewhat vague.

Answer: I corrected the sentence. Lines 66-67. I also added new content in the introduction section. Lines: 34-37.

Sample Size and Generalizability:

Answer: I added the limitation that the study was conducted in only one city in lines 247-252 and also provided a suggestion for future study.

Questions/Areas for Further Research and future direction:

Answer: Given the limitations, I have provided suggestions for future articles. Lines: 247-253, 261-264.
